# Comparison of Mental Health and Quality of Life Symptom Networks in Adolescents Exposed and Not Exposed to Cyberbullying: Evidence from Chinese High School Students

**DOI:** 10.3390/bs15111498

**Published:** 2025-11-04

**Authors:** Yanzhe Zhang, Yushun Han, Kaiyu Guan

**Affiliations:** 1 Northeast Asian Studies College, Jilin University, Changchun 130012, China; yanzhe_zhang@jlu.edu.cn (Y.Z.); hanys22@mails.jlu.edu.cn (Y.H.); 2 Institute of Mental Health, Peking University, Beijing 100083, China

**Keywords:** cyberbullying, network analysis, mental health, quality of life, adolescents

## Abstract

With the widespread use of the internet, cyberbullying has become a significant issue affecting adolescents’ mental health and quality of life. This study utilized propensity score matching (PSM) and network analysis to compare the mental health and quality of life symptom networks of Chinese high school students who had experienced cyberbullying and those who had not. A total of 9066 students were assessed using the Symptom Checklist (SCL-90) and the Chinese Quality of Life Scale for Primary and Secondary School Students (QLSCA). Network comparison tests revealed significant structural differences (M = 0.2136, *p* < 0.05), with the cyberbullying group showing higher global network strength (11.985 vs. 10.700, *p* < 0.05), indicating a more densely connected symptom network. In both groups, “self-satisfaction” was the most central node, but the cyberbullying group exhibited higher centrality for “negative emotion” and “self-concept” compared to anxiety and depression in the non-cyberbullying group. Key bridging symptoms differed: “academic attitude” in the non-cyberbullying group and “opportunity for activity” in those who had experienced cyberbullying. Moreover, the connection strength between “interpersonal sensitivity” and “negative emotion” was stronger in the cyberbullying group. These findings suggest that targeted interventions should focus on emotional regulation and social activity to disrupt the symptom network cycle.

## 1. Introduction

With the widespread use of digital devices, the internet has become deeply embedded in adolescents’ daily lives, social interactions, and learning activities (Anthony et al., 2022). Cyberbullying is of particular concern in this virtual environment (Prkno et al., 2025). Cyberbullying refers to aggressive behaviours carried out through electronic media, which are repeated and intended to harm others (Bottino et al., 2015; Patchin & Hinduja, 2015). Compared to traditional bullying, cyberbullying is more likely to be overlooked by parents and schools, yet it may lead to profound and severe psychological and social functional impairments (Wiederhold, 2024). Empirical studies have consistently shown that cyberbullying is widespread among adolescent populations (Eyuboglu et al., 2021; Fahy et al., 2016). Of note, during the COVID-19 pandemic, lockdowns resulted in teens spending a lot more time in front of screens, which put a strain on their mental health (Ravens-Sieberer et al., 2021; Singh & Balhara, 2021). This, in turn, further heightened both the risk and the detrimental impact of cyberbullying.

In China, with the internet penetration rate among minors reaching an exceptionally high level, adolescents are widely engaged in online life. According to the China Internet Network Information Centre, 193 million adolescents used the internet in 2022, resulting in an internet penetration rate of 97.2%. Of these users, 97.5% were in cities, 96.5% were in rural areas, 95.1% were in primary school, and over 99% were in all other school-age groups (Youth League Central Committee, 2023). Over the past decade, Chinese adolescents have experienced a significant surge in internet penetration, reflecting a profound shift in digital engagement. The use of the internet has played an important role in addressing the issue of adolescents’ access to learning and social interaction. However, of particular concern is the emerging risk of cyberbullying (Chen et al., 2023).

There is a large body of literature on the association between experiences of cyberbullying and a range of adverse mental health outcomes. These outcomes include, but are not limited to, depressive symptoms (Chang et al., 2013; Fahy et al., 2016), anxiety (Ravens-Sieberer et al., 2021), diminished self-esteem, self-harm behaviours, and suicidal ideation and attempts (Bottino et al., 2015; Nagata et al., 2025). Moreover, cyberbullying has been shown to seriously impair adolescents’ quality of life (QoL), a comprehensive health indicator that encompasses physical, psychological, social, and environmental dimensions (Yin et al., 2024). Several studies have shown that compared with their non-bullied peers, adolescents who experience cyberbullying report significantly lower QoL (McLoughlin et al., 2022).

Traditional paradigms in psychopathology research, such as latent variable models, have long conceptualized symptoms such as depression and anxiety as interchangeable indicators or outward manifestations of an underlying disorder (e.g., major depressive disorder). This approach emphasizes the calculation of the total symptom score as a measure of disorder severity, which, although clinically valuable, has been criticized for failing to address the complex interrelationships that may exist among symptoms. For adolescents, an important and much-debated question is whether cyberbullying leads to a more “tightly connected” or “rigid” symptom network, in which problems mutually reinforce each other, generating a vicious cycle that hinders recovery. Within this trauma-induced network, it is not yet clear which psychological symptoms and QoL dimensions occupy the most central positions. A comprehensive understanding of the underlying mechanisms through which cyberbullying impacts the mental health and QoL of Chinese adolescents is essential for formulating efficacious prevention and intervention strategies.

In recent years, the network analysis approach in psychopathology has provided a new and revolutionary perspective on the nature of psychological symptoms. This approach no longer treats symptoms as mere interchangeable indicators of an underlying latent construct, but rather conceptualizes psychopathology itself as a complex system of interrelated symptoms (Zhang et al., 2024). Each symptom in this system is called a “node”, and the statistical links between symptoms are called “edges”. By constructing and visualizing such psychological networks, researchers are able to demonstrate the complex patterns of interrelationships among symptoms and identify the nodes that play key roles within the network. For instance, core symptoms—those that are most strongly linked to others and have the most impact—are thought to be very important in initiating and maintaining the symptom network (Rai et al., 2025). Theoretically, targeting these central nodes for intervention may be more effective in dismantling the overall symptom network and thus, achieving better treatment outcomes.

Cyberbullying, as a prevalent form of online aggression among adolescents, has been consistently linked to multiple adverse mental health outcomes, including depression, anxiety, and diminished life satisfaction (Ravens-Sieberer et al., 2021; Rodriguez-Rivas et al., 2022). Adolescents exposed to cyberbullying often experience overlapping psychological and social difficulties that interact in complex ways, making them an important population for network-based psychological analysis. However, when comparing adolescents exposed to cyberbullying with those not exposed, a key methodological challenge is the presence of confounding variables. The likelihood of experiencing cyberbullying and an adolescent’s psychological health may be simultaneously influenced by factors such as gender, age, and family relationships. Without adequate control for these confounders, the true impact of cyberbullying may be misestimated.

Although research on cyberbullying and mental health has grown rapidly, few studies have systematically applied a network analytic framework to explore the interconnections among mental health symptoms and quality of life (QoL) indicators—particularly in the Chinese adolescent population (Agustiningsih et al., 2024). Chinese adolescents face unique academic pressures and cultural expectations that may interact with cyberbullying to shape their psychological symptoms and QoL networks. Existing international studies have primarily examined symptom networks or QoL networks separately and often without statistically controlling for confounding variables. To date, no study in China has directly compared the mental health and QoL symptom networks between adolescents exposed and not exposed to cyberbullying after controlling for confounders using PSM (Yin et al., 2024).

Building upon the above evidence and theoretical considerations, the present study aimed to address two key research questions. First, do adolescents exposed to cyberbullying exhibit different network characteristics (e.g., global connectivity, network density, and edge strength) in mental health (SCL-90) and QoL (QLSCA) compared to those not exposed? Second, which symptom or QoL domains demonstrate higher centrality within each group’s network, and how do these central nodes differ between adolescents exposed and not exposed to cyberbullying?

To answer these questions, the present cross-sectional study combined Propensity Score Matching (PSM) and network analysis to investigate the structural characteristics of mental health and QoL networks among Chinese adolescents exposed and not exposed to cyberbullying. PSM was used to match participants between groups on key demographic and family variables, thereby minimizing potential confounding effects. After matching, subscale-level data (rather than item-level data) from the Symptom Checklist-90 (SCL-90) and the Quality of Life Scale for Children and Adolescents (QLSCA) were used to construct and compare psychological and QoL networks. The analysis focused on differences in global network structure, edge strength, and centrality to identify the most influential psychological and QoL domains associated with cyberbullying exposure.

## 2. Materials and Methods

### 2.1. Study Design and Setting

Hosted by the National Population Health Data Centre (Shengfa et al., 2022), the data for this study were derived from the publicly accessible longitudinal database known as the “China Adolescent Health Database” (DYH). This database is designed to offer long-term monitoring of the health conditions and associated behavioural patterns of Chinese adolescents. The present study employed cross-sectional survey data collected in Shandong Province during the 2020–2021 academic year. A sampling procedure proportionate to population size was adopted to ensure that the sample was representative in terms of geographical distribution, population size, and socioeconomic status.

### 2.2. Participants

The final sample consisted of 9066 high school students, 4378 boys and 4688 girls. Prior to the implementation of the study, all potential participants and their legal guardians were informed of the study’s goals, processes, privacy rules, and how the data would be used. The research has been approved by the Ethics Committee of Shandong University, China.

### 2.3. Measures

#### 2.3.1. Cyberbullying

Cyberbullying was assessed using a single direct question regarding participants’ experiences over the past year. The question was: “In the past 12 months, have you ever experienced cyberbullying (e.g., being insulted, threatened, excluded, or attacked by rumours through text messages, QQ, WeChat, Weibo, or other social media platforms)?” The response options were “Yes” or “No.” Based on each participant’s response, they were classified into a “cyberbullying group” (answered “Yes”) and “non-cyberbullying group” (answered “No”).

#### 2.3.2. Mental Health

Mental health symptoms were measured by the Chinese version of the internationally recognized Symptom Checklist-90 (SCL-90) (Fan et al., 2024). The SCL-90 is a classic instrument for evaluating individual mental health status and is particularly suitable for screening a wide range of symptoms. Comprising 90 items, the questionnaire requires participants to evaluate each item based on their genuine emotions experienced during the preceding week, with responses measured on a five-point Likert scale ranging from 1 (“not at all”) to 5 (“extremely”). The SCL-90 encompasses nine fundamental dimensions of symptomatology: somatization, obsessive-compulsive symptoms, interpersonal sensitivity, depression, anxiety, hostility, phobic anxiety, paranoid ideation, and psychoticism. Each subscale measures a different symptom, and higher scores indicate more severe symptoms (Zhu & Xue, 2025). Although the SCL-90 was originally developed for adults, it has also been applied to adolescents. In particular, the instrument has been widely used among Chinese adolescents with satisfactory reliability and validity (Yang et al., 2024). In the present network analysis, nodes were labelled according to these subscales, denoted as SCL1 to SCL9. The internal consistency of the SCL-90 in this sample was excellent, with a Cronbach’s α of 0.968.

#### 2.3.3. Quality of Life

Quality of Life was assessed using the Chinese Quality of Life Scale for Children and Adolescents (QLSCA), which was locally developed to evaluate the subjective QoL of Chinese children and adolescents. The QLSCA consists of 49 items, which ultimately form four factors and 13 dimensions: (1) Social-Psychological Functioning (SPF), which includes relationships between teachers and students, between peers, between parents and children, learning ability and attitudes, and self-concept; (2) Physical-Mental Health (PMH), which includes physical sensations, negative emotions, and attitudes toward schoolwork; (3) Living Environment (LE), which includes things like how easy it is to get around, activities that are available, and exercise; and (4) Satisfaction with Life Quality (SLQ), which measures how happy a person is with themselves and other parts of their life overall. A five-point Likert scale is used to rate each item. Higher scores indicate better QoL. In this study, subscale totals were used as indicators of each dimension. In the network analysis, nodes were labelled according to these subscales, denoted as QOL1 to QOL13. The internal consistency of the QLSCA was also high, with a Cronbach’s α of 0.865.

#### 2.3.4. Covariates

Additionally, the questionnaire collected demographic data such as age and gender, as well as family-related factors such as household registration type, only-child status, and paternal frequent intoxication, for inclusion as covariates in the PSM analysis. Prior studies have demonstrated that these factors are intricately linked to both the mental health and bullying behaviours exhibited by adolescents (Reynales-Shigematsu et al., 2024).

### 2.4. Statistical Analyses

First, to reduce the influence of confounding factors on the study results, PSM was conducted. A logistic regression model was used to determine each participant’s propensity score. The experience of cyberbullying was the dependent variable, and age, gender, type of residence, and family environment were the independent variables. Based on a calliper set at 0.2 of the standard deviation, 1:1 nearest-neighbour matching was then performed. This ensured that each person in the cyberbullying group was matched with the person in the non-cyberbullying group who had the closest likelihood score (R. Wang et al., 2024).

Second, symptom network models of mental health and QoL were constructed separately for the cyberbullying and non-cyberbullying groups. Gaussian Graphical Models (GGMs) were used to figure out the structure of the network because all factors were continuous. The graphical LASSO method was used to determine the partial correlation matrix between the nodes in more detail. LASSO is a regularization method that punishes the model by reducing weak edges to zero. These edges are likely to be caused by sampling error, and they make the network model sparse, stable, and easy to understand (Epskamp et al., 2018). The Extended Bayesian Information Criterion (EBIC) was used to find the best regularization parameter. The adjusting parameter (γ) was set to 0.5. Many studies have indicated that this setting is good because it strikes a good mix between sensitivity (finding real edges) and specificity (not showing false edges) (Li et al., 2025; S. Wang et al., 2025). Taking into account both positive and negative connections, the Expected Influence (EI) of a node was computed as the aggregate sum of all edge weights. According to (Robinaugh et al., 2016), a higher EI suggests that the node assumes a more pivotal position within the network structure. The Bridge Expected Influence (BEI), which indicates the influential role of a node as a bridge symptom connecting communities, was calculated by summing the edge weights between a specific node and nodes in the other community, with a higher BEI value implying a more significant bridging function (Jones et al., 2019).

In order to evaluate the precision and reliability of the network, multiple procedures were carried out. Initially, to determine the precision of the edge weights, nonparametric bootstrapping was employed, with 95% confidence intervals (CIs) calculated for enhanced accuracy. Narrow bootstrapped CIs suggest that sampling variability in the edge weights is minimal, indicating a more accurate network estimation. To investigate whether the centrality order was preserved across different subsets of data, the stability of node centrality was subsequently evaluated through a subset bootstrap method incorporating case-dropping. To quantify the stability of the centrality indices, the correlation stability (CS) coefficient was utilized, which is defined as the maximal proportion of cases that can be removed while maintaining highly correlated values (r > 0.7). A CS coefficient exceeding 0.25 reflects acceptable stability, while values surpassing 0.5 are deemed to represent outstanding stability (Epskamp et al., 2018). A bootstrap differential test was performed alongside 1000 permutations to investigate possible differences in network features.

Finally, a Network Comparison Test (NCT) was carried out. The NCT method was used to carefully assess whether statistically significant differences existed between the two networks. This test relies on permutation testing principles, and the null distribution was constructed using 2000 resamples in this study. To evaluate possible differences, three tests were conducted: invariance of network structure, comparison of global strength, and analysis of edge strength (van Borkulo et al., 2023). Symptom networks with numerous edges often face challenges when strict multiple-comparison corrections are applied to all connections, as this can lead to an excessively conservative significance threshold, significantly raising the likelihood of Type II errors, where actual differences may remain undetected. Therefore, to control for Type I errors while preserving sufficient statistical power, no formal multiple-comparison correction was implemented for the edge-difference tests. However, a more rigorous significance threshold was implemented, with the *p*-value set below 0.001.

### 2.5. Ethical Considerations

The study was conducted in accordance with the Declaration of Helsinki and approved by the Ethics Committee of Shandong University, China (protocol code 20180517). Written informed consent was obtained from all participants and their legal guardians before participation.

## 3. Results

### 3.1. Participant Descriptive Statistics Before and After PSM

Table 1 provides a comprehensive overview of the sample characteristics before PSM. A total of 9066 high school students were included in this study, with 766 reporting experiences of cyberbullying and 8300 reporting no such experiences. Several demographic characteristics exhibited significant differences between the two groups. Specifically, the cyberbullying group exhibited a significantly higher mean age compared to the non-cyberbullying group, with respective values of 16.23 ± 1.25 and 16.13 ± 1.24 years (mean ± SD) (*p* = 0.027). Furthermore, in the cyberbullying group, there were significantly more girls (54.2% vs. 45.8%, *p* < 0.001), and most participants were not only children (71.2% vs. 57.3%, *p* < 0.001). Furthermore, there were statistically significant differences between the two groups in terms of household registration type and family relationships, suggesting potential sociodemographic influences.

In order to mitigate the potential bias arising from these baseline differences, the PSM method was employed in this study. Following the matching process, a total of 766 students in the cyberbullying group were successfully matched on a 1:1 basis with 766 students in the non-cyberbullying group. Following the matching procedure, no statistically significant disparities in demographic characteristics persisted between the two groups (Table 2).

We further present detailed comparisons of the scores on each dimension of the mental health and QoL measures between the two matched groups. Independent-samples *t*-tests revealed that, in comparison to the non-cyberbullying group, the cyberbullying group demonstrated significantly higher scores across all nine psychological symptom dimensions (all *p* < 0.001). Teenagers exposed to cyberbullying tended to exhibit more pronounced symptoms across multiple dimensions, including physical complaints, obsessive behaviours, social insecurity, depressive moods, anxious feelings, aggressive tendencies, phobic reactions, paranoid thoughts, and psychotic traits. The cyberbullying group demonstrated markedly lower scores across all 13 QoL dimensions (*p* < 0.001 for each). These results provide clear evidence that experiences of cyberbullying victimization are significantly and closely linked to poorer mental health and reduced QoL.

### 3.2. Network Structure

**Figure 1:**
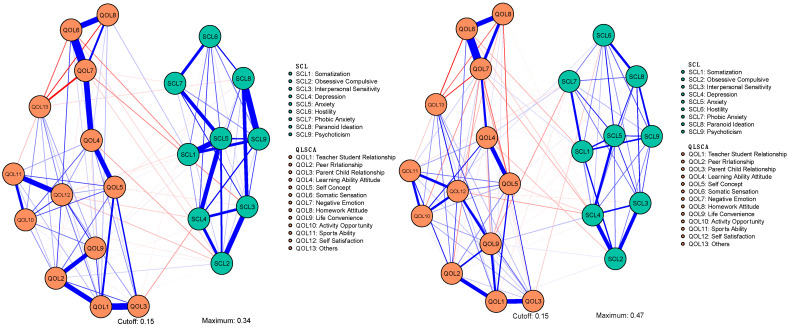
Network structures of mental health and quality of life symptoms in the non-cyberbullying group (**left**) and the cyberbullying group (**right**).

To examine the network structures related to mental health and QoL, regularized networks were constructed based on the scores from all subscales. The two networks had 109 and 125 edges with non-zero weights, out of a total of 231 possible connections. Figure 1 illustrates the network structures of both the non-cyberbullying and cyberbullying groups, where Figure 1(left) corresponds to the non-cyberbullying group and Figure 1(right) to the cyberbullying group. In the non-cyberbullying group, SCL4 (“Depression”) showed the highest predictability, achieving an R^2^ value of 0.900. In the cyberbullying group, SCL5 (“Anxiety”) showed the highest predictability, with an R^2^ value of 0.922.

### 3.3. Centrality Estimation

**Figure 2:**
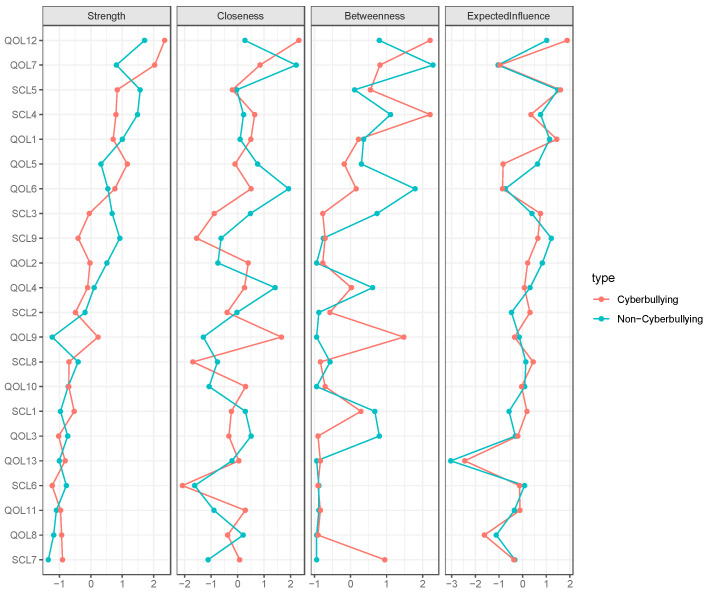
Comparisons of the centrality measures between the non-cyberbullying group and the cyberbullying group.

**Figure 3:**
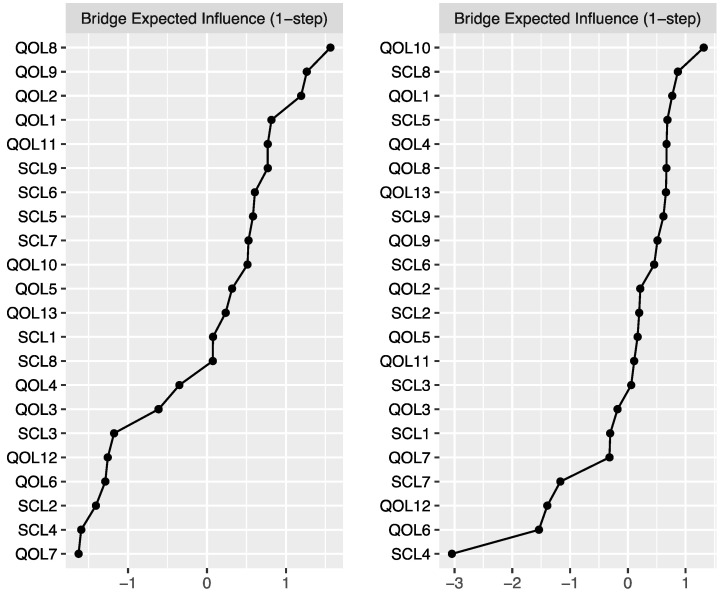
Bridge expected influence in the non-cyberbullying group (**left**) and the cyberbullying group (**right**).

Figure 2 presents the centrality measures for both groups, demonstrating the relative significance of each symptom within the network structures. In both groups, QOL12 (“Self Satisfaction”) demonstrated the most significant centrality. In the non-cyberbullying group, SCL5 (“Anxiety”) and SCL4 (“Depression”) emerged as the next most central symptoms, while in the cyberbullying group, QOL7 (“Negative Emotion”) and QOL5 (“Self Concept”) were the next most pivotal indicators. In the non-cyberbullying group, the three nodes with the highest expected influence (EI) values were SCL5 (‘Anxiety’, EI = 1.49), SCL9 (‘Psychoticism’, EI = 1.21), and QOL1 (‘Teacher–Student Relationship’, EI = 1.14), each exerting a substantial impact within the network (Figure 3). In contrast, in the cyberbullying group, the top three nodes were QOL12 (‘Self Satisfaction’, EI = 1.88), SCL5 (‘Anxiety’, EI = 1.60), and QOL1 (‘Teacher–Student Relationship’, EI = 1.45). These findings indicate that, although certain symptoms like Anxiety and Teacher–Student Relationship represent core features in both groups, the impacts of other symptoms, such as Psychoticism within the non-cyberbullying group and Self-Satisfaction among the cyberbullying group, fluctuate based on the extent of exposure to cyberbullying experiences. In the non-cyberbullying group, the key bridge symptom was QOL8 (“Homework Attitude”), whereas in the cyberbullying group, it was QOL10 (“Activity Opportunity”).

### 3.4. Estimation of Network Accuracy and Stability

**Figure 4:**
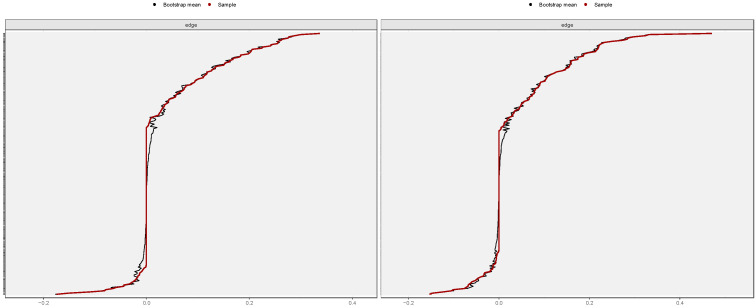
Bootstrapped 95% confidence intervals of the estimated edge weights in the non-cyberbullying group (**left**) and the cyberbullying group (**right**).

**Figure 5:**
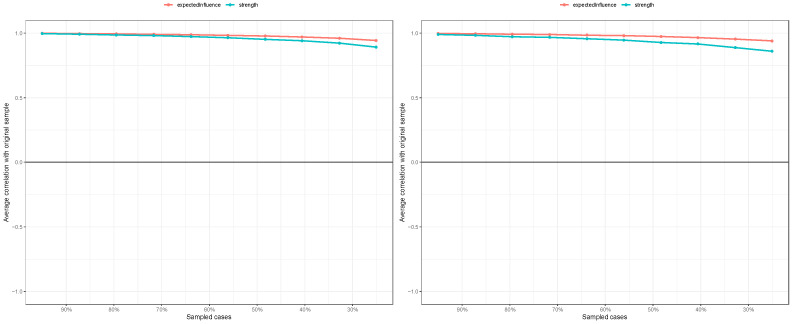
Centrality stability for the mental health symptoms network in the non-cyberbullying group (**left**) and the cyberbullying group (**right**).

**Figure 6:**
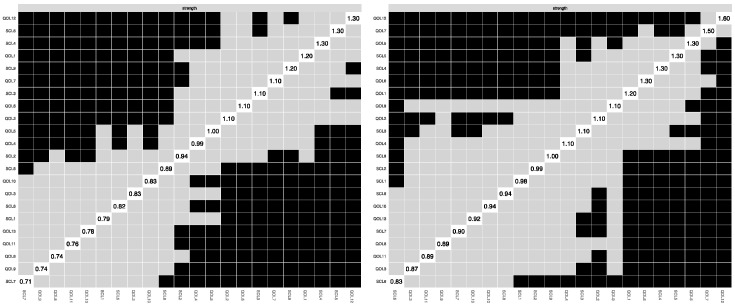
Bootstrapped difference test for expected influence in the non-cyberbullying group (**left**) and the cyberbullying group (**right**).

**Figure 7:**
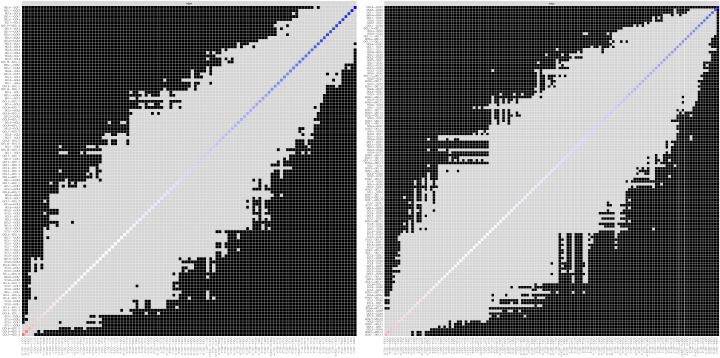
Bootstrapped difference test for edge weights. The **left** figure shows the non-cyberbullying group, and the **right** figure shows the cyberbullying group.

Figure 4 illustrates the accuracy of the edge weight estimation through the bootstrap method for both the non-cyberbullying group and the cyber-bullying group. As shown in the figure, both networks exhibited strong stability. The CS coefficients (Figure 5) indicate that, for both the non-cyberbullying and cyberbullying groups, the centrality indices were identical—0.749 for strength and 0.749 for expected influence—both exceeding the recommended threshold of 0.5. Figure 6 and Figure 7 present the results of the tests examining the differences in strength centrality and expected influence.

### 3.5. Network Comparison

**Figure 8:**
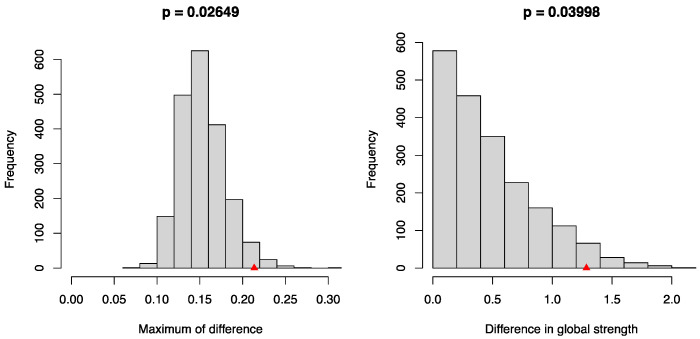
Network comparison between the non-cyberbullying group and the cyberbullying group. The left panel shows the distribution of the maximum differences in network structure invariance, and the right panel shows the distribution of differences in global strength. *p*-values were calculated as the proportion of permuted groupings (2000 permutations) that yielded differences at least as extreme as the observed values.

The NCT was used to assess the differences in the mental health and QoL symptom networks between the cyberbullying and non-cyberbullying groups. The initial test for network structure invariance revealed significant disparities in the overall structural configuration between the cyberbullying group and the non-cyberbullying group (M = 0.2136, *p* < 0.05), thereby suggesting the presence of distinct and statistically significant structural differences between these two groups (Figure 8, left). Subsequently, the global strength in the cyberbullying group was found to be significantly higher than in the non-cyberbullying group (global strength: 11.985 versus 10.700, *p* < 0.05), suggesting that mental health and QoL were more densely interconnected in the cyberbullying group (Figure 8, right). The findings also revealed that, in comparison to the non-cyberbullying group, the cyber-bullying group demonstrated significantly elevated EI in two specific nodes—SCL2 (“Obsessive Compulsive”) and QOL13 (“Others”)—whereas QOL5 (“Self Concept”) exhibited a markedly lower level of EI. Additionally, two edges differed significantly between the two groups (Table 3): SCL3 (“Interpersonal Sensitivity”) and QOL7 (“Negative Emotion”), and QOL6 (“Somatic Sensation”) and QOL7 (“Negative Emotion”). The connection between SCL3 (“Interpersonal Sensitivity”) and QOL7 (“Negative Emotion”), as well as between QOL6 (“Somatic Sensation”) and QOL7 (“Negative Emotion”), was stronger in the cyberbullying group than in the non-cyberbullying group.

## 4. Discussion

To the best of our knowledge, this study represents the first attempt to employ a network analytic approach to systematically compare the differences in mental health and quality of life (QoL) symptom networks between adolescents exposed and not exposed to cyberbullying in China. By applying Propensity Score Matching (PSM) to control for key confounders, this study addresses a critical research gap identified in the Introduction—namely, the lack of network-based comparative evidence under a statistically balanced design (Lee et al., 2025; Prkno et al., 2025). The findings revealed that adolescents exposed to cyberbullying exhibited more densely connected networks, as well as distinctive core and bridge symptoms, thereby extending existing research by illustrating how cyberbullying exposure may relate to complex interconnections among psychological and QoL domains within adolescent populations.

The most important finding of the present study is that the symptom network in the cyberbullying group exhibited significantly higher global strength. In network theory, a more tightly connected network is often considered a more “rigid” or “vulnerable” system (Yin et al., 2024). Once a node such as “Anxiety” is activated, its influence rapidly spreads throughout the network, leading to a chain reaction of other issues, such as “Depression” and “Decline in Self-Concept”, which traps the individual in a psychological state that is difficult to recover from. This suggests that the psychological symptoms and QoL issues of cyberbullying victims are more likely to trigger and maintain each other, creating a vicious cycle that is difficult to break. This high connectivity may explain why the negative effects of cyberbullying tend to be pervasive and long-lasting (Lee et al., 2025).

Regarding core symptoms, the networks of both groups displayed considerable similarities as well as notable differences, with “Self Satisfaction” (QOL12) showing the highest centrality in each group. This underscores the pivotal role of self-assessment in adolescent overall well-being. However, in the cyberbullying group, the centrality of “Self Satisfaction” was particularly pronounced. This aligns with the characteristic of cyberbullying, often targeting an individual’s self-worth through demeaning and humiliating behaviours (Prkno et al., 2025). When self-satisfaction is compromised, it may become a key driver of other mental health issues, such as depression and interpersonal sensitivity, as well as a decline in QoL, such as deteriorating peer relationships. “Anxiety” (SCL5) and “Teacher-Student Relationship” (QOL1) were also important central nodes in both groups, reflecting the universal impacts of academic stress and school relationships on the psychological state of all adolescents. Interestingly, in the cyberbullying group, the centrality of “Negative Emotion” (QOL7) and “Self Concept” (QOL5) increased, replacing “Depression” (SCL4) and “Psychoticism” (SCL9) in the non-cyberbullying group. This suggests that, for victims, widespread negative emotional experiences and unstable self-concept have become key driving factors within their psychological networks, potentially reflecting direct emotional and cognitive responses to sustained cyber harm (Campbell et al., 2012).

The differences in bridge symptoms reveal key pathways through which mental health issues interact with QoL concerns. In the non-cyberbullying group, “Attitude toward School” (QOL8) served as a key bridge, indicating that, for most adolescents, especially high school students, academic attitudes are the primary link between their internal world and QoL. However, in the cyberbullying group, “Activity Opportunity” (QOL10) emerged as the most significant bridge symptom. This finding is particularly insightful. Cyberbullying often leads to social withdrawal and reduced participation in activities (Moura Carias et al., 2024), and the lack of opportunities for engaging in beneficial physical and social activities further exacerbates feelings of loneliness and negative emotions, making recovery from the trauma of bullying more difficult. Therefore, the reduction in “Activity Opportunity” may serve as a crucial mediator in translating the internal psychological distress caused by cyberbullying (e.g., depression, anxiety) into a broader decline in QoL (e.g., social isolation, lower life satisfaction).

Furthermore, the differences in the connection strength of specific edges provide profound insights into the mechanisms of cyberbullying’s impact. In the cyberbullying group, the connection between “Interpersonal Sensitivity” (SCL3) and “Negative Emotion” (QOL7) was significantly strengthened. This finding is entirely consistent with the nature of cyberbullying as an interpersonal attack (Tokunaga, 2010). Victims become highly vigilant and sensitive to others’ evaluations and online interactions, and this heightened sensitivity often triggers intense negative emotions, creating a reinforced cycle of “hyper-vigilance-emotional distress”. Similarly, the enhanced connection between “Somatic Sensation” (QOL6) and “Negative Emotion” (QOL7) highlights the process of somatization of psychological distress (Hutson et al., 2017). Adolescents who experience cyberbullying are more likely to transform inexpressible emotional stress into physical discomfort, a common stress response in this age group (Schneider et al., 2012).

Consistent with a substantial body of existing literature (Kowalski et al., 2014), the findings in this study identify cyberbullying as a significant risk factor that contributes to adolescent mental health problems. A substantial body of prior research has consistently revealed significant correlations between cyberbullying victimization and various psychological outcomes, including depression (Maurya et al., 2022), anxiety disorders (Stea et al., 2024), diminished self-esteem (Ejaz & Khaliq, 2025), as well as suicidal ideation (Hinduja & Patchin, 2010). This study, through the analysis of a large-scale sample of Chinese adolescents, further verifies the universality of these associations while considering potential moderating factors.

This study has several notable strengths. First, the study utilized data from a large and representative sample, while the application of PSM successfully balanced the two groups in terms of key demographic and family background variables. This contributes to significant improvement in the accuracy and robustness of the research findings. Second, to the best of our knowledge, this is among the few studies to apply network analysis to investigate the impact of cyberbullying, moving beyond traditional approaches that primarily focus on linear associations between variables. By adopting a network perspective, the present study provides a more dynamic and systemic understanding of the complexity of psychopathology. Finally, by integrating mental health symptoms (SCL-90) and QoL life dimensions (QLSCA) within a single network model, we were able to identify bridging symptoms linking these two domains, which offers novel insights for developing more targeted intervention strategies.

The findings of this study have important practical implications. First, the highly connected network structure observed in the cyberbullying group suggests that interventions for victims must be comprehensive and implemented rapidly to prevent the spread and consolidation of symptoms. Interventions targeting a single symptom (e.g., depression) may be of limited effectiveness; instead, multi-faceted approaches addressing emotion regulation, self-concept, and interpersonal relationships are required. Second, the centrality analysis identified “self-satisfaction”, “negative emotion”, and “anxiety” as core symptoms. These symptoms should be prioritized as intervention targets. Interventions focusing on these symptoms—such as programs to enhance self-esteem and self-confidence, as well as training in emotion regulation—may generate broader beneficial effects by dismantling the pathological network as a whole. Finally, the identification of “activity opportunity” as a bridging symptom highlights the importance of behavioural activation and social support. Schools, families, and communities should collaborate to ensure that adolescents exposed to cyberbullying are not isolated from positive social activities. Encouraging participation in sports, arts, or extracurricular clubs may represent a crucial intervention strategy for disrupting the link between psychological distress and diminished QoL (Wasserman et al., 2023).

While this study provides valuable insights, several limitations should be noted. First, due to its cross-sectional design, causal inferences cannot be drawn. Although cyberbullying is associated with differences in network structures, certain network characteristics (e.g., high connectivity) may also increase vulnerability or intensify related psychological symptoms. Future longitudinal research is needed to clarify these bidirectional dynamics. Second, although PSM controlled for demographic and family variables, other potential confounders—such as offline bullying, academic stress, and screen time—were not included. Future studies should account for these contextual factors to achieve a more comprehensive understanding of adolescents’ mental health. Third, cyberbullying exposure was measured using a single binary item, limiting information on frequency and severity. Employing multi-item or frequency-based tools would enhance measurement precision (Schneider et al., 2012). Finally, the sample was limited to high school students from Shandong Province, China; thus, caution is warranted when generalizing the findings to other populations or cultural contexts. Additionally, network metrics should be interpreted with caution, as high centrality does not necessarily indicate therapeutic priority or clinical relevance. Future studies should examine whether highly central symptoms identified in network models represent effective intervention targets (Borsboom et al., 2018).

## 5. Conclusions

In conclusion, the data were derived from a cross-sectional survey conducted in a single province (Shandong, China) during the 2020–2021 academic year. Using network analysis, this study examined the associations between cyberbullying victimization and the interconnections among mental health and quality of life (QoL) symptoms in adolescents. Adolescents exposed to cyberbullying demonstrated denser and more interconnected symptom networks, suggesting stronger psychological interdependence among mental health and QoL domains. Self-satisfaction and negative emotions emerged as central nodes, while activity opportunities acted as a bridge linking psychological distress with diminished QoL. These findings enhance our understanding of the complex associations between cyberbullying exposure and adolescents’ well-being and may help inform more comprehensive prevention and support strategies. However, caution is warranted when generalizing these findings, as cyberbullying exposure was assessed using a dichotomous (yes/no) item, and the study design does not permit causal inference. Future research should replicate these findings with longitudinal, multi-regional data and more detailed measures of cyberbullying experiences.

## Figures and Tables

**Table 1:** Descriptive characteristics of the participants before propensity score matching.

Variable	Non-Cyberbullying (*n* = 8300)	Cyberbullying (*n* = 766)	*p*-Value
Age (year), mean (sd)	16.13 ± 1.24	16.23 ± 1.25	0.027
Sex, n (%)			<0.001
Girls	4041 (48.7)	415 (54.2)	
Boys	4259 (51.3)	351 (45.8)	
Household registration type			0.022
This city	4041 (48.7)	415 (54.2)	
This township	3451 (41.6)	293 (38.3)	
Other cities	529 (6.4)	39 (5.1)	
Other province	279 (3.4)	19 (2.5)	
Only child			<0.001
No	4041 (48.7)	415 (54.2)	
Yes	4259 (51.3)	351 (45.8)	
Father drunkenness			<0.001
No	7483 (90.2)	646 (84.3)	
Yes	817 (9.8)	120 (15.7)	

**Table 2:** Descriptive characteristics, mental health, and quality of life of the participants after propensity score matching.

Variable	Non-Cyberbullying (*n* = 766)	Cyberbullying (*n* = 766)	*p*-Value
Age (year), mean (sd)	16.21 ± 1.22	16.23 ± 1.25	0.756
Sex, n (%)			1
Girls	341 (44.5)	341 (44.5)	
Boys	425 (55.5)	425 (55.5)	
Household registration type			0.999
This city	415 (54.2)	415 (54.2)	
This township	292 (38.1)	293 (38.3)	
Other cities	39 (5.1)	39 (5.1)	
Other province	20 (2.6)	19 (2.5)	
Only child			0.918
No	436 (56.9)	439 (57.3)	
Yes	330 (43.1)	427 (42.7)	
Father drunkenness			1
No	647 (84.5)	657 (84.3)	
Yes	119 (15.5)	120 (15.7)	
Somatization	15.98 (6.29)	21.41 (10.18)	<0.001
Obsessive–compulsive	16.64 (6.85)	20.09 (9.04)	<0.001
Interpersonal sensitivity	13.83 (5.94)	17.62 (8.37)	<0.001
Depression	19.06 (8.28)	25.08 (12.26)	<0.001
Anxiety	14.36 (6.21)	18.82 (9.27)	<0.001
Hostility	8.15 (3.35)	11.16 (5.65)	<0.001
Phobic anxiety	9.32 (3.94)	12.43 (6.25)	<0.001
Paranoid ideation	8.40 (3.46)	11.14 (5.42)	<0.001
Psychoticism	13.80 (5.45)	18.18 (8.51)	<0.001
Teacher student relationship	16.15 (2.82)	13.52 (4.14)	<0.001
Peer relationship	15.96 (2.85)	13.54 (4.10)	<0.001
Parent child relationship	12.32 (2.53)	10.45 (3.28)	<0.001
Learning ability attitude	7.97 (1.61)	7.30 (1.47)	<0.001
Self concept	10.80 (2.56)	9.63 (3.04)	<0.001
Somatic sensation	14.62 (2.94)	13.42 (3.91)	<0.001
Negative emotion	11.00 (2.46)	10.48 (3.04)	<0.001
Homework attitude	8.66 (1.72)	8.16 (2.23)	<0.001
Life convenience	6.69 (1.24)	5.69 (1.80)	<0.001
Activity opportunity	7.83 (2.10)	6.94 (2.44)	<0.001
Sports ability	8.39 (2.22)	7.29 (2.49)	<0.001
Self Satisfaction	17.86 (3.66)	15.14 (4.76)	<0.001
Others	5.44 (1.02)	5.04 (1.54)	<0.001

**Table 3:** Edge invariance test between the non-cyberbullying group and the cyberbullying group (*p* < 0.001).

Edge Between		*p*-Value
SCL3	QOL7	0.0009
QOL6	QOL7	0.0009

## Data Availability

The data used in this study were obtained from the Population Health Data Archive (PHDA) of the National Population Health Data Center (https://www.ncmi.cn).
